# Perfluorooctane sulfonate exerts inflammatory bowel disease-like intestinal injury in rats

**DOI:** 10.7717/peerj.10644

**Published:** 2021-01-08

**Authors:** Hai Liang, Miao Yang, Cheng Zeng, Wei Wu, Liying Zhao, Yu Wang

**Affiliations:** 1 Department of Pharmacy, The People’s Hospital of Bozhou, Bozhou, Anhui Province, China; 2 Department of Neurology, The People’s Hospital of Bozhou, Bozhou, Anhui Province, China; 3 Department of Pharmacy, Deqing People’s Hospital, Huzhou, Zhejiang Province, China; 4 Department of Pharmacy, Zhejiang Integrated Traditional and Western Medicine Hospital, Hangzhou, Zhejiang Province, China

**Keywords:** Inflammatory bowel disease, Perfluorooctane sulfonate, Pathological change, Apoptosis, Inflammatory infiltration

## Abstract

**Background:**

Perfluorooctane sulfonate (PFOS), a type of perfluorinated compounds (PFCs), can induce various organ toxicity, including hepatomegaly, immunotoxicity, and gut microbiota disorder. PFCs have been associated with inflammatory bowel disease (IBD). Yet, whether PFOS exposure causes IBD-like disorder and the underlying mechanism remains undefined. Here, we investigated the influence of PFOS exposure on the development of IBD-like disorder in rats.

**Methods:**

Sprague-Dawley rats were intraperitoneally injected with PFOS (1 or 10 mg/kg) or normal saline (NS) every other day for 15 days. Body weight, serum concentrations of serum amyloid A (SAA) and high sensitivity C reactive protein (hsCRP) were measured. Pathological assessments of villi height and crypt depth in the proximal duodenum and jejunum were performed using H&E staining. Terminal deoxynucleotidyl transferase dUTP nick end labeling (TUNEL) staining was used to assay cell apoptosis in the jejunum. The infiltration of inflammatory cells and cytokines in the jejunum were detected by immunohistochemistry analysis.

**Results:**

PFOS (10 mg/kg) significantly increased the body weight, SAA and hsCRP, whereas no significant differences were observed in PFOS 1 mg/kg group of rats. The villi height and crypt depth in the proximal duodenum and jejunum were significantly reduced upon PFOS exposure. PFOS induced higher histopathological score in intestinal tissues compared to NS. Notably, TUNEL-positive cells were significantly higher in the jejunum upon PFOS exposure. Further, neutrophil and macrophage accumulated, and inflammatory cytokines infiltration were also remarkably increased in rats exposed to PFOS.

**Conclusion:**

PFOS induces IBD-like phenotypes in rats, with associated inflammatory infiltration to intestinal.

## Introduction

Inflammatory bowel disease (IBD) is a chronic and incurable disorder, a result of a disordered immune response and environment triggered in the susceptible host (Flynn & Eisenstein, 2019). The morbidity and prevalence of IBD is increasing considerably worldwide, especially in developing countries (Ng et al., 2018). In general, this disorder can affect individuals of all ages. Although information on the etiopathogenesis of IBD is limited, attention have been paid to the increased frequency of IBD due to exposure to xenobiotics (Miller et al., 2012).

As a rising class of bioaccumulative contaminants and persistent pollutants, perfluorinated compounds (PFCs) have been ubiquitously found in organisms and the environment. PFCs, peculiarly perfluorooctane sulfonate (PFOS) and perfluorooctanic acid (PFOA), have been widely applied in industry and consumer products, from lubricants and adhesives to nonstick coatings (DeWitt et al., 2012; Ferrari et al., 2019). PFCs can cause extensive pollution because of their widespread existence in air, water, soil, plant and even in the tissues of wildlife and humans (Liu et al., 2020). Notably, PFCs have long half-life once absorbed into the blood, ranging from several weeks to a few years in humans (Olsen et al., 2007). Studies have shown that both professional and nonprofessional exposure sources contribute to the body burden of PFOS for people (Chang et al., 2020; Wang et al., 2018). Available data from the Environmental Protection Agency (EPA) on the US population exposure to PFCs have been estimated as 6 million individuals affected by PFOS, PFOA from public drinking water at levels above the 70 pg/ml dose recommended by the EPA (Hu et al., 2016). There are restrictive regulations relating production and usage of polyfluoroalkyl compounds set out by international organizations (UNEP, 2016). However, PFCs are still available in China and in most Asian market (Xie et al., 2013).

General toxicological studies on experimental animals exposed to PFOS have revealed reproduction toxicity, hepatomegaly, neurotoxicity, and immunotoxicity (Zhang et al., 2014; Liang et al., 2019; Wang et al., 2019; Qazi et al., 2009; Zheng et al., 2009). For example, in vivo PFOS treatment induced atrophy of spleen and thymus (Qazi et al., 2009), and inhibited Th1 responses, while promoting Th2 responses (Zheng et al., 2009). PFOS treatment also caused hepatic metabolic dysfunction, which strongly correlated with alterations in microbiota composition (Zhang et al., 2020). As a long half-life pollutant, PFOS can accumulate in the intestine, and improperly influence the enteral homeostasis under physiological and pathological conditions. C57BL/6J mice orally exposed to PFOS exhibited damaged microbiota and short-chain fatty acids, with modulated barrier function of the gut environment (Wang et al., 2020).

Several studies have reported that PFCs are associated with IBD (Steenland et al., 2013; Steenland, Zhao & Winquist, 2015). A clinical research involving 32,000 individuals from a C8 community found that serum PFOA concentration positive correlated with ulcerative colitis, a kind of IBD (Steenland et al., 2013). In this cohort, there is an increased risk of developing into ulcerative colitis (Steenland, Zhao & Winquist, 2015). On the contrary, a retrospective study showed no obvious evidence supporting PFOS and PFHxS yearly exposure as a risk factor for IBD (Xu et al., 2020). Thus, animal studies are needed to explore the effects of PFCs on IBD and related pathological changes of the intestines.

Preliminary data have shown that PFCs have a substantial impact on inflammatory responses. In wild bottlenose dolphins, a positive correlation was demonstrated between plasma levels of PFOS and PFOA with clinical parameters of inflammation (Fair et al., 2013). Futher, PFOS elevated inflammatory cytokines expression and reduced mucin production in a bacterial infection mice model (Suo et al., 2017). TNF-α and IL-6, two markers of inflammation, were reportedly modulated by PFOS exposure *in vivo* and *in vitro* studies (Han et al., 2018). However, the mechanism that underlies inflammation responses caused by PFOS in intestinal tract is still unclear.

In the present study, we explored whether PFOS exposure causes IBD-like damages in the proximal jejunum and duodenum, and whether the pathological changes are associated with inflammatory infiltration in rats.

## Materials & Methods

### Animals

Male Sprague-Dawley rats (220 ± 5 g) were purchased from the Laboratory Animal Center of Zhejiang Province (Hangzhou, China). All rats were housed in an environmentally-controlled room (22−26 °C, 12 h light-dark cycle, relative humidity 60%), and had free access to standard chow and water. Animals received humane care in accordance with the National Institutes of Health Guide for the Care and Use of Laboratory Animals. The animal experiments were accredited by the Ethics Committee of Laboratory Animal Care and Welfare, Zhejiang Academy Medical Sciences, approval number 2018-142.

### Experimental protocols

PFOS was purchased from Sigma Aldrich company (MO, USA), and dissolved in dimethylsulfoxide (DMSO) to obtain a 100 mg/ml stock solution. After a one-week adjustable feeding period, the rats were assigned to three groups of six rats: two experimental groups and one control group. Rats in the experimental groups were intraperitoneally injected PFOS at 1 mg/kg and 10 mg/kg every other day for 15 days. PFOS was diluted with normal saline (NS). Rats in the control group received an equal volume of NS. The dose and period of PFOS were selected according to previous studies (Wen et al., 2016; Lau et al., 2003). The body weight in each group were weighted before and after PFOS treatment. At the end of the treatment, rats were anesthetized (S-ketamine 100 mg/kg, and diazepam 1.5 mg/kg) and sacrificed. Blood, the duodenum, and the jejunum were harvested for biochemical determination or histology.

### Blood biochemical

Blood samples were collected from the aorta abdominalis in anesthetized rats. Samples were left to stand at room temperature for 1 h, at 4 ° C for 2 h, and subsequently centrifuged at 3,000 g for 10 min. The supernatant was harvested and stored at −80 ° C for blood biochemical indexes determination. The serum amyloid A (SAA) protein and high sensitivity C reactive protein (hsCRP) were quantified by using commercial enzyme-linked immunosorbent assay (ELISA) kits (USCN Life Science, Wuhan, China) according to the manufacturer’s instructions.

### Histopathological analysis

The proximal duodenum and proximal jejunum were obtained from three groups for histopathological analysis. Samples were fixed in 4% formalin with phosphate buffer solution (PBS), and were embedded in paraffin. Two 5 µm slides of each rats were cut and stained with hematoxylin and eosin (H&E) for analysis. Images were taken at 200 × and 100 ×using a light microscope (Leica Microsystems, Wetzlar, Germany). All crypts were performed at 200 ×, and depth of crypts was measured by Image-Pro Plus software version 6.0 (Media Cybernetics, MD, USA). Intestinal villus was captured at 100 ×, and the height of villus was assessed.

Histopathological score of intestinal tissues were performed based on the appearance and severity of lesions according to a previously described method (Lucioli et al., 2013). The tissue score used a scale of 0-12 points, where numbers of crypts and villus, the height of villus, the morphological change of enterocytes, the extent of villus coalescence, and pathological injury of tissues including autolysis, edema, apoptosis and necrotic debris were inferred. Scoring of morphological and injury data was implemented by two blinded observers.

### Immunohistochemical analysis

Inflammatory infiltration was evaluated through immunohistochemical analysis of intestinal tissues. Sections from each groups were blocked using 5% goat serum and subsequently incubated in specific primary antibodies, including F4/80 (LS-C96373-100, Lifespan, 1:1000 dilution), Ly6g (GTX40912, GeneTex, 1:500 dilution), IL-6 (ab9324, Sigma, 1:1000 dilution), and TNF-α (ab6671, Abcam, 1: 600 dilution). Slides then were washed thrice with PBS and stained with horseradish peroxidase-conjugated secondary antibodies. Substrate DAB was applied for visualization. Images were obtained under high-power fields with a microscope.

### TUNEL analysis

Apoptosis was assessed by terminal deoxynucleotidyl transferase dUTP nick end labeling (TUNEL) staining (In Situ Cell Death Detection Kit, POD (Roche)). TUNEL assay was performed in accordance with the manufacturer’s instructions. TUNEL-positive cells were quantitated per small-intestinal tissue slide per rat using the Image-Pro Plus software. Three random fields were selected in each slide, and the values were averaged.

### Statistical analysis

All data are presented as the mean ± standard deviation (SD). Statistical significance (*p* < 0.05) was analyzed using one-way analysis of variance (ANOVA) followed by Newman-Keuls multiple comparison test. Data analyses were performed with GraphPad Software (Prism Version 8.01).

## Results

### Changes in body weight and inflammatory markers in PFOS exposed rats

**Figure 1:**
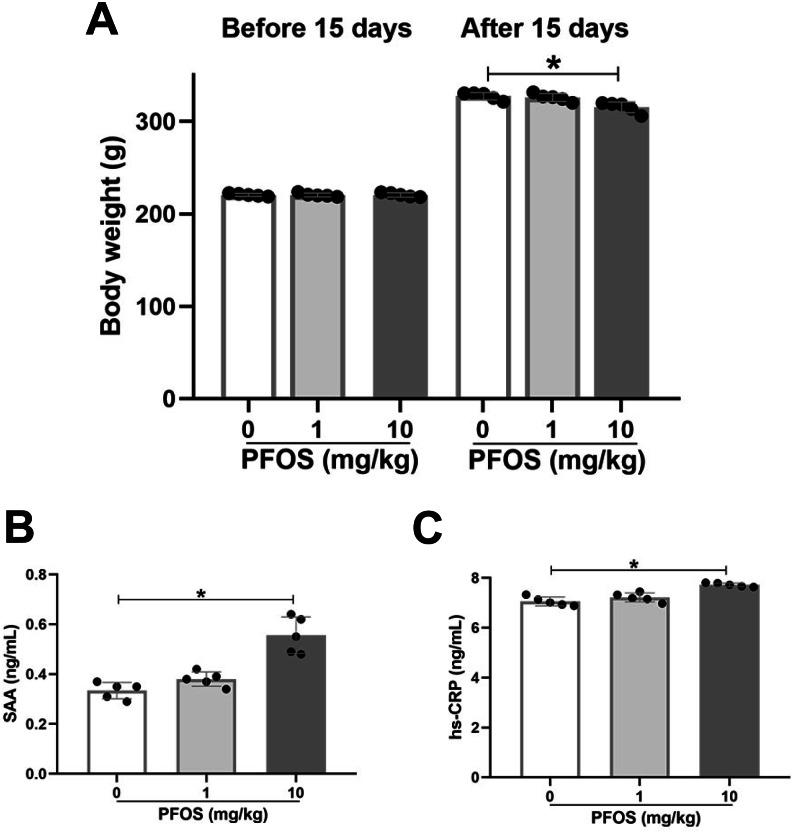
Effect of PFOS on body weight and inflammatory markers in rats. SD rats received intraperitoneal injection of PFOS with 0 mg/kg (normal saline), 1 mg/kg and 10 mg/kg every other day for 15 days. (A) Body weight was measured before and after PFOS treatment. The serum amyloid A (SAA) protein (B) and high sensitivity C reactive protein (hsCRP; C) were quantified by ELISA assay. SAA and hsCRP were significantly increased in rats treated with 10 mg/kg PFOS compared with 0 mg/kg PFOS group. Values are mean ± SD, *N* = 5/group. *P < 0.05.

Sprague-Dawley rats were exposed to 0 mg/kg (NS), 1 mg/kg, and 10 mg/kg PFOS every other day for 15 days. Body weight was measured before and after 15 days, and inflammatory markers, such as SAA and hsCRP, were detected in serum. Exposure to 10 mg/kg PFOS significantly lowered rats’ body weights compared with 0 mg/kg PFOS (315.2 ± 5.9 *vs* 327.4 ± 3.9, *P* < 0.05), whereas there was no significant difference between the PFOS 1 mg/kg and PFOS 0 mg/kg rats groups (*P* > 0.05, Fig. 1A). As shown in Figs. 1B and 1C, 10 mg/kg PFOS was sufficient to increase serum SAA and hsCRP compared with PFOS 0 mg/kg (SAA: 0.56 ± 0.07 *vs* 0.33 ± 0.03 ng/ml, *P* < 0.05; hsCRP: 7.72 ± 0.08 *vs* 7.06 ± 0.18 ng/ml, *P* < 0.05). Levels of SAA and hsCRP in rats exposed to 1 mg/kg PFOS were slightly elevated compared with those in the PFOS 0 mg/kg group, although no significant differences were observed (*P* > 0.05, Figs. 1B, 1C).

To evaluate the effects of subchronic PFOS exposure on SAA and hsCRP levels, rats were administered PFOS by gavage once two days for 15 days and 28 days. In the PFOS 10 mg/kg group, both the 15-day and 28-day exposures presented significantly increased levels of hsCRP and SAA compared with the PFOS 0 mg/kg groups (*P* < 0.05, Figs. S1A, S1B). Levels of hsCRP and SAA in the PFOS 1 mg/kg groups were not significant difference compared with the PFOS 0 mg/kg groups (*P* > 0.05, Figs. S1A, S1B).

### PFOS exposure induces histological lesions in the proximal duodenum and proximal jejunum of rats

**Figure 2:**
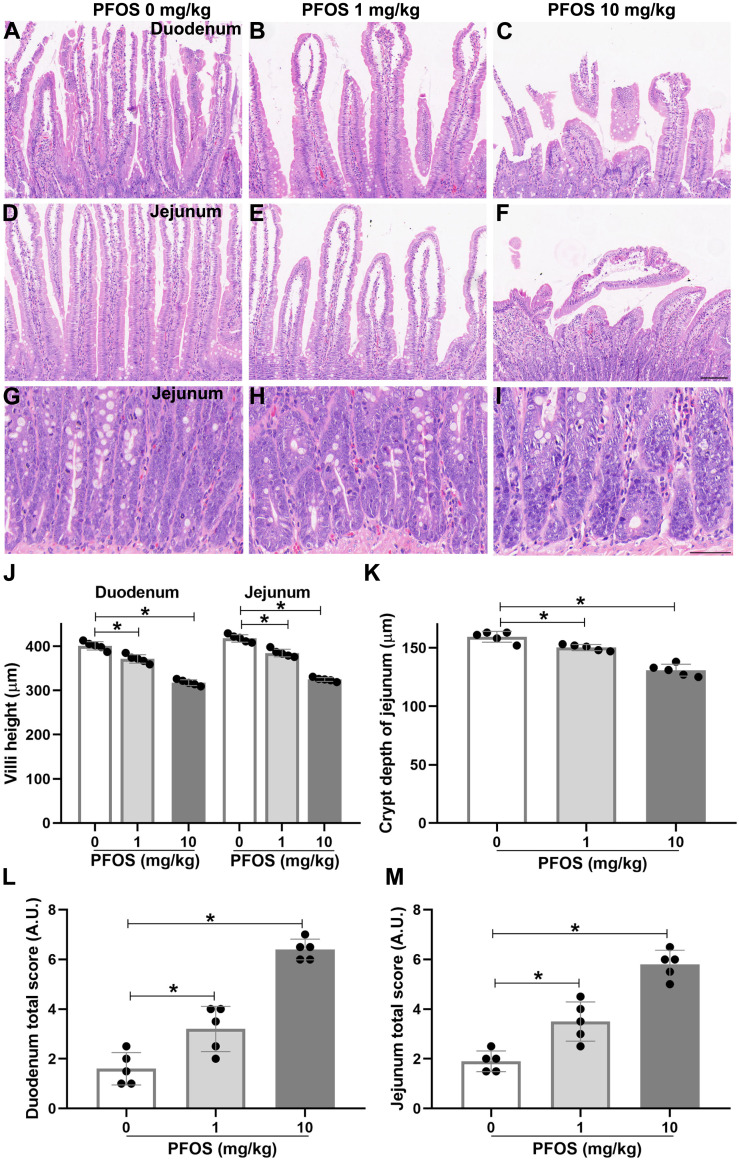
PFOS-induced histological lesions in the proximal duodenum and proximal jejunum of rats. After 15 days of PFOS treatment, intestinal tissues of rats were collected and fixed for H&E staining. (A–F) Representative images of the villi from the duodenum and jejunum were shown. Villi with normal morphology were exhibited in PFOS 0 mg/kg group, and villi with different degrees of coalescence and autolytic were observed in PFOS 1 mg/kg and 10 mg/kg groups. Bar 100 µm, 100 ×. (G–I) Representative images of the crypt from the jejunum were shown. Crypt with deformity in PFOS 1 mg/kg and 10 mg/kg group. Bar 50 µm, 400 ×. Villi height (J) and crypt depth (K) were analyzed. Lesion scores of the duodenum (L) and the jejunum (M) were assessed after histological examination according to the extent of severity. Values are mean ± SD, *N* = 5/group. *P < 0.05.

To determine whether PFOS exposure could induce susceptibility to IBD-like injury in rats, we used an intestinal toxicity model of PFOS and detected histological changes in proximal duodenum and jejunum by H&E staining. H&E sections of the duodenum and jejunum presented obviously coalescent, autolytic, and shorter villi in the PFOS 10 mg/kg group, compared with the PFOS 0 mg/kg group (Figs. 2A, 2C, 2D, 2F, 2J). Similar results were observed with 28 days of 10 mg/kg PFOS exposure (Figs. S1C, S1E, S1F, S1H, S1I). The PFOS 1 mg/kg group showed decreased heights of villi in the duodenum and jejunum (Figs. 2A, 2B, 2D, 2E, 2J). Exposure to 1 mg/kg PFOS for 28 days also resulted in a significant reduction of villi height (Figs. S1D, S1F, S1G, S1I). Irregularly shaped crypts with reduced depth was also exhibited in the jejunum of the PFOS 1 mg/kg and 10 mg/kg groups compared with the PFOS 0 mg/kg group (Figs. 2G–2D, 2K). Further, duodenum and jejunum morphological scores were significantly elevated in the PFOS 1 mg/kg and 10 mg/kg groups (Figs. 2L, 2M). Similar results were observed in both groups exposed to 1 mg/kg and 10 mg/kg PFOS for 28 days (Figs. S1J). These observations suggest that PFOS exposure can promote dose-dependent histological lesions in the duodenum and jejunum, with consequent development of IBD-like intestinal dysfunction.

### PFOS exposure induces cell apoptosis in jejunum of rats

**Figure 3:**
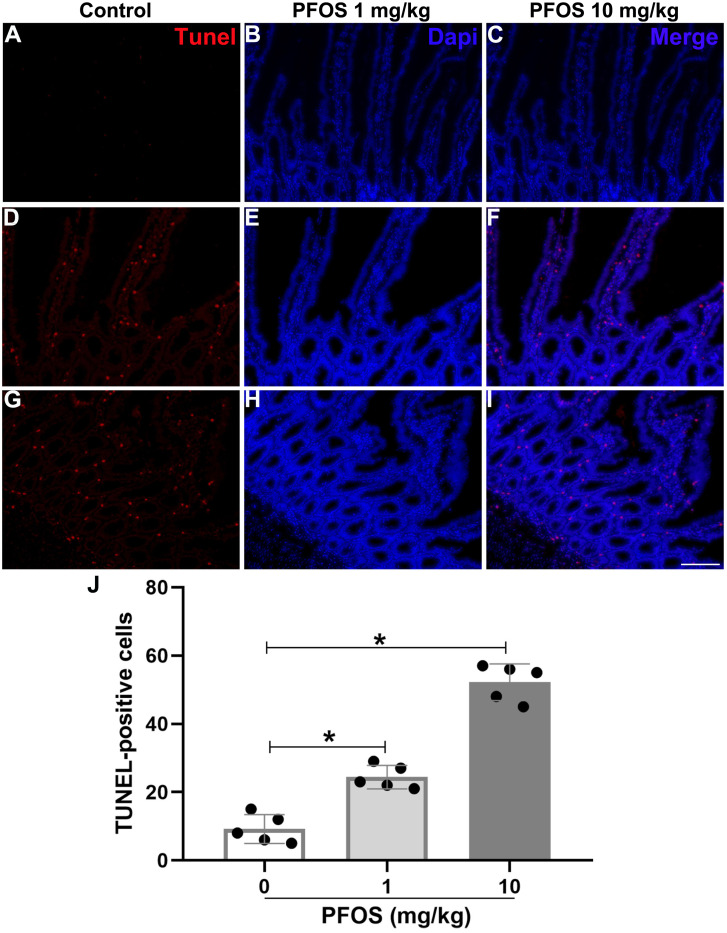
PFOS-induced cell apoptosis in the proximal jejunum of rats. (A–I) Apoptotic cells were determined by terminal deoxynucleotidyl transferase dUTP nick end labeling (TUNEL) staining. Positive red staining for TUNEL; blue staining for dapi. Bar 100 µm, 200 ×. (J) TUNEL positive cells were counted in different groups. PFOS exposure with different dose significantly increased apoptotic cells compared with 0 mg/kg PFOS group. Values are mean ± SD, *N* = 5/group. *P < 0.05.

To investigate the influence of PFOS exposure on cell apoptosis in the jejunum of rats, TUNEL staining was performed. Both 1 mg/kg group and 10 mg/kg PFOS upregulated the number of TUNEL-positive cells in the jejunum compared with 0 mg/kg PFOS (PFOS 1 mg/kg group: 24.4 ± 3.4 *vs* 9.2 ± 4.2, *P* < 0.05; PFOS 10 mg/kg group: 52.2 ± 5.4 *vs* 9.2 ± 4.2, *P* < 0.05, Figs. 3A–3J).

### PFOS exposure induced inflammation infiltrated in jejunum of rats

**Figure 4:**
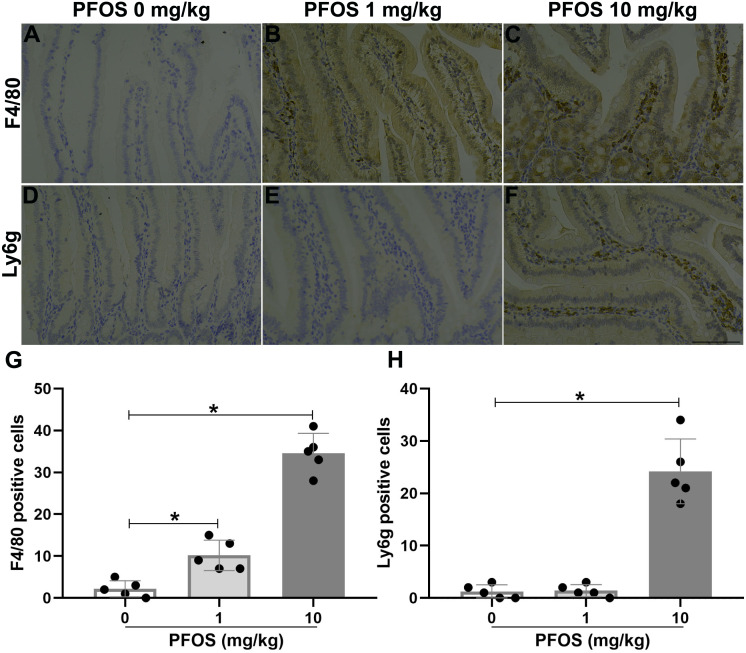
Effect of PFOS exposure on inflammatory cell infiltration in the proximal jejunum of rats. (A–C) Immunohistochemistry staining in jejunum showed the effect of PFOS exposure on macrophage accumulation. Brown staining for F4/80, a marker of macrophage. (D–F) Representative images of the neutrophil infiltration were shown. Brown staining for Ly6g, a marker of neutrophil. Bar 100 µm, 200×. (G–H) Statistical graphs. PFOS exposure with 1 mg/kg and 10 mg/kg significantly induced macrophage and neutrophil infiltration in the jejunum of rats. Values are mean ± SD, *N* = 5/group. *P < 0.05.

**Figure 5:**
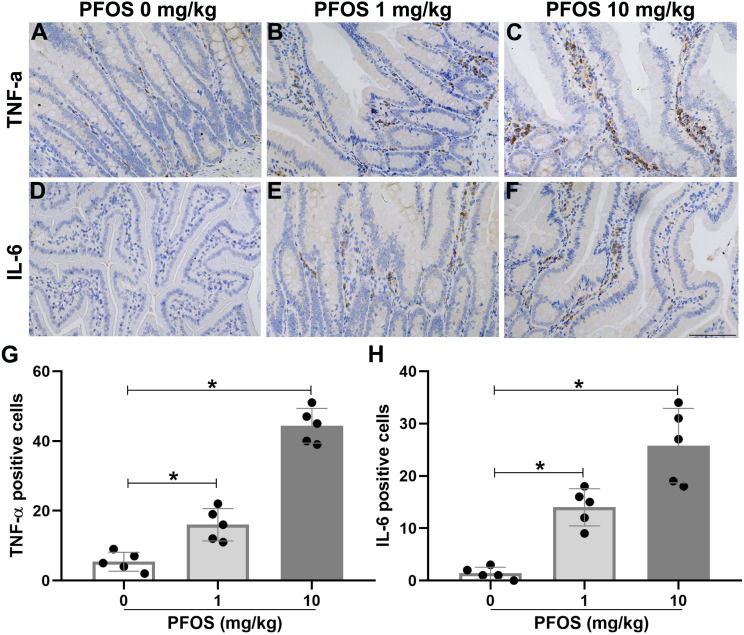
PFOS exposure induced inflammatory factors infiltration in the proximal jejunum of rats. (A–F) Immunohistochemistry staining in jejunum showed PFOS exposure on TNF- *α* and IL-6 infiltration. Brown staining for TNF-*α* or IL-6. Bar 100 µm, 200×. (G–H) Protein levels of TNF-α and IL-6 were analyzed. PFOS exposure greatly enhanced levels of TNF-α and IL-6 compared with PFOS 0 mg/kg group. Values are mean ± SD, *N* = 5/group. *P < 0.05.

To investigate the effect of PFOS exposure on jejunal inflammation in rats, we detected the infiltration of inflammatory cells and inflammatory factors. As shown in Figs. 4A–4C and 4G, immunohistochemistry experiments revealed more macrophages accumulation, indicated by F4/80, within the jejunum of the PFOS 1 mg/kg and 10 mg/kg groups than in the PFOS 0 mg/kg group (PFOS 1 mg/kg group: 10.2 ± 3.6 *vs* 2.2 ± 1.9, *P* < 0.05; PFOS 10 mg/kg group: 34.6 ± 4.7 *vs* 2.2 ± 1.9, *P* < 0.05). Similarly, neutrophil infiltration, indicated by Ly6g, within the jejunum was markedly increased in the PFOS 10 mg/kg group compared with PFOS 0 mg/kg group (24.2 ± 6.2 *vs* 1.2 ± 1.3, *P* < 0.05, Figs. 4E, 4F, 4H). Notably, there was no significant increase in neutrophil infiltration in the jejunum of the PFOS 1 mg/kg group (*P* > 0.05, Figs. 4E, 4G, 4H). In agreement with the PFOS-induced increase in macrophage and neutrophil infiltration, there was also a marked upregulation of proinflammatory cytokines TNF-α and IL-6 expression in the jejunum of the PFOS 1 mg/ml and 10 mg/ml groups compared with the PFOS 0 mg/ml group (TNF-α: PFOS 1 mg/kg group: 16.0 ± 4.6 *vs* 5.4 ± 2.7, *P* < 0.05; PFOS 10 mg/kg group: 44.4 ± 5.0 *vs* 5.4 ± 2.7, *P* < 0.05, Figs. 5A–5C, 5G; IL-6: pFOS 1 mg/kg group: 14.0 ± 3.5 *vs* 1.4 ± 1.1, *P* < 0.05; PFOS 10 mg/kg group: 25.8 ± 7.1 *vs* 1.4 ± 1.1, *P* < 0.05, Figs. 5D–5F, 5H). Collectively, these results indicate that PFOS exposure induces IBD-like lesions associated with an upregulated pro-inflammatory infiltration.

## Discussion

In the present study, we provided evidence that PFOS exposure induced IBD-like injury, reflected by an increase in SAA and hsCRP levels, and histological lesions of villi and crypts in the duodenum and jejunum of rats. This damage is involved in PFOS-induced upregulation of cell apoptosis and inflammatory infiltration. We integrated these findings in a toxic rat model via PFOS exposure doses.

In patients with IBD, as well as autoimmune enteropathy patients, studies have been largely descriptive of a shortened life expectation and poignantly worse quality of life (Chapel et al., 2008; Kelsen, Russo & Sullivan, 2019). IBD, as one of the most common autoimmune diseases, includes ulcerative colitis and Crohn’s disease. Crude prevalence for diagnosed cases per 100,000 individuals over the age of 20 were 410 for ulcerative colitis and 300 for Crohn’s disease (Cooper, Bynum & Somers, 2009). So far, few environmental risk factors for IBD have been identified. A crucial factor is smoking, which has been strongly associated with Crohn’s disease (Mahid et al., 2006). Oral contraceptive use and prior gastrointestinal infections have also been positively associated with ulcerative colitis, whereas appendictis and breastfeeding are protective factors for the disorder (Ordas et al., 2012). Although the documented findings on the effect of environmental pollution in IBD is limited, attentions have been raised on the potential increase in the morbidity of IBD with increased exposure to xenobiotics. PFOS and PFOA are ubiquitous xenobiotics in the serum of US residents, and has been shown to affect immune responses in rodents (Zheng et al., 2009; Steenland et al., 2013).

Due to their particular physical and chemical capabilities, PFCs are widely used in industry that produces everyday items, some of which are persistently present in the environment, wildlife, and even humans (Liu et al., 2020). PFOS, as one of the principal PFCs, has been detected in human blood, and its average half-life period is almost 5.4 years (Olsen et al., 2007). The large body of evidence has shown that PFOS accumulation in animals results in cardiotoxicity, hepatotoxicity, immune deficiency, reproductive, and developmental toxicity (Harada et al., 2005; Zheng et al., 2009; Liang et al., 2019). For example, in a guinea-pig model, PFOS exposure led to a reduction in the action potential duration of ventricular myocytes (Harada et al., 2005). A study in children aged 5 and 7 years old revealed a close relationship between decreased humoral immune response and PFOS exposure (Grandjean et al., 2012). Although several researchers have attempted to elucidate possible mechanisms underlying PFOS-induced organ toxicity, additional studies are needed to validate the effect and related mechanisms of PFOS exposure in intestinal injury.

There was a positive association between accumulative PFOA, another fluorocarbon, and the incidence of ulcerative colitis among 32,254 American adults (Steenland et al., 2013). Notably, previous research has focused on PFOS influence on gut microbiota and host metabolism (Wang et al., 2020; Zhang et al., 2020; Steenland et al., 2013; Steenland, Zhao & Winquist, 2015). Thus, information about PFOS in relation to pathological injury of small intestine in rodents is insufficient. In this study, we found that 10 mg/kg PFOS treatment for 15 days greatly lowered the body weight of rats, which was consistent with a previous study in rats orally exposed to high dose of PFOS for 28 days (Han et al., 2018). SAA and hsCRP are the most commonly used biomarkers of IBD in the clinic and laboratory (Jergens et al., 2003). We found that PFOS (10 mg/kg) exposure significantly increased serum SAA and hsCRP levels. In consistency with this observation, we also found that PFOS 10 mg/kg induced histopathological lesions in the proximal duodenum and jejunum, reflected by reduction of villi height and crypt depth. There was also a slight pathological change in the proximal duodenum and jejunum of the PFOS 1 mg/kg group. Further, PFOS induced upregulation of apoptotic cell induced in the jejunum of exposed rats.

Inflammation can disrupt the maintenance and homeostasis of the intestinal tract. IBD is commonly characterized by transmural inflammation, especially neutrophil inflammation (Kelsen, Russo & Sullivan, 2019). Several studies have reported the inflammatory response caused by PFOS exposure (Wang et al., 2020; Gong et al., 2019). Wang et al. (2020) have found that a short-term exposure to low doses of PFOS induced intestinal barrier damage, which led to peripheral inflammation that exacerbated liver injury in mice. Furthermore, PFOS exposure in rats caused severe inflammatory cell infiltration in the liver and upregulated serum IL-6 and TNF-α levels (Han et al., 2018). In the present study, we found that macrophage and neutrophil were upregulated in the jejunum upon PFOS 10 mg/kg exposure. However, part of the macrophage infiltration was also observed in the PFOS 1 mg/kg group. Activation of macrophages could promote overexpression of pro-inflammatory cytolines, such as IL-6 and TNF-α, which in turn activates macrophage infiltration to tissues such as, liver and intestine (Jurjus et al., 2015). Obviously, this is a vicious circle of inflammatory responses, with pathological change in tissues being the overall outcome. Our results showed that TNF-α and IL-6 were overexpressed in the jejunum of rats upon PFOS exposure (1 mg/kg and 10 mg/kg). Therefore, we suggest that PFOS-induced inflammatory response is most likely associated with the small intestinal toxicity.

## Conclusions

In the current study, our results indicate that PFOS exposure causes increase in serum levels of SAA and hsCRP, and pathological injury of villi and crypt in the duodenum and jejunum of rats. These lesions are likely involved in PFOS-induced aggravation of cell apoptosis and inflammatory response, specifically including inflammatory cells infiltration and TNF-α and IL-6 overexpression. Our results suggest that individuals living in environments containing PFOS may exhibit dysfunction intestinal physiology that could trigger IBD-like disorder. The effective therapies are needed to improve the toxicity in animals and humans exposed to this pollutant.

##  Supplemental Information

10.7717/peerj.10644/supp-1 Supplemental Information 1 Effect of PFOS exposure on inflammatory markers and histological lesions in the proximal duodenum and proximal jejunum of rats Rats were administrated with PFOS by gavage once two days for 15 days or 28 days. The doses of PFOS were 0 mg/kg (normal saline), 1 mg/kg and 10 mg/kg. After PFOS treatment, blood and intestinal tissues of rats were collected for biochemical determination and H&E staining respectively. The high sensitivity C reactive protein (hsCRP; A) and serum amyloid A (SAA) protein (B) were measured by ELISA assay. (C-H) Representative images of the villi from the duodenum and jejunum were shown. Bar 100 µm, 100 ×. (I) Villi height were analyzed. (J) Lesion scores of the duodenum and the jejunum were assessed. Values are mean ± SD, *N* = 5/group. *P < 0.05.

10.7717/peerj.10644/supp-2 Supplemental Information 2 Raw data.

10.7717/peerj.10644/supp-3 Supplemental Information 3 Supplementary material.

## Additional Information and Declarations

### Data Availability

The following information was supplied regarding data availability: Raw data are available in the Supplementary File.

## References

[ref-1] Chang S, Parker GA, Kleinschmidt SE, Olsen GW, Ley CA, Taiwo OA (2020). A pathology review of the lower gastrointestinal tract in relation to ulcerative colitis in rats and cynomolgus macaques treated with ammonium perfluorooctanoate. Toxicologic Pathology.

[ref-2] Chapel H, Lucas M, Lee M, Bjorkander J, Webster D, Grimbacher B, Fieschi C, Thon V, Abedi MR, Hammarstrom L (2008). Common variable immunodeficiency disorders: division into distinct clinical phenotypes. Blood.

[ref-3] Cooper GS, Bynum ML, Somers EC (2009). Recent insights in the epidemiology of autoimmune diseases: improved prevalence estimates and understanding of clustering of diseases. Journal of Autoimmunity.

[ref-4] DeWitt JC, Peden-Adams MM, Keller JM, Germolec DR (2012). Immunotoxicity of perfluorinated compounds: recent developments. Toxicologic Pathology.

[ref-5] Fair PA, Romano T, Schaefer AM, Reif JS, Bossart GD, Houde M, Muir D, Adams J, Rice C, Hulsey TC, Peden-Adams M (2013). Associations between perfluoroalkyl compounds and immune and clinical chemistry parameters in highly exposed bottlenose dolphins (Tursiops truncatus). Environmental Toxicology and Chemistry.

[ref-6] Ferrari F, Orlando A, Ricci Z, Ronco C (2019). Persistent pollutants: focus on perfluorinated compounds and kidney. Current Opinion in Critical Care.

[ref-7] Flynn S, Eisenstein S (2019). Inflammatory bowel disease presentation and diagnosis. Surgical Clinics of North America.

[ref-8] Gong X, Yang C, Hong Y, Chung ACK, Cai Z (2019). PFOA and PFOS promote diabetic renal injury in vitro by impairing the metabolisms of amino acids and purines. Science of the Total Environment.

[ref-9] Grandjean P, Andersen EW, Budtz-Jørgensen E, Nielsen F, Mølbak K, Weihe P, Heilmann C (2012). Serum vaccine antibody concentrations in children exposed to perfluorinated compounds. Journal of the American Medical Association.

[ref-10] Han R, Zhang F, Wan C, Liu LM, Zhong Q, Ding WJ (2018). Effect of perfluorooctane sulphonate-induced kupffer cell activation on hepatocyte proliferation through the NF-*κ*B/TNF-α/IL-6-dependent pathway. Chemosphere.

[ref-11] Harada K, Xu F, Ono K, Iijima T, Koizumi A (2005). Effects of PFOS and PFOA on L-type Ca2+ currents in guinea-pig ventricular myocytes. Biochemical and Biophysical Research Communications.

[ref-12] Hu XC, Andrews DQ, Lindstrom AB, Bruton TA, Schaider LA, Grandjean P, Lohmann R, Carignan CC, Blum A, Balan SA, Higgins CP, Sunderland EM (2016). Detection of poly-and perfluoroalkyl substances (PFASs) in U.S. drinking water linked to industrial sites, military fire training areas, and wastewater treatment plants. Environmental Science & Technology Letters.

[ref-13] Jergens AE, Schreiner CA, Frank DE, Niyo Y, Ahrens FE, Eckersall PD, Benson TJ, Evans R (2003). A scoring index for disease activity in canine inflammatory bowel disease. Journal of Veterinary Internal Medicine.

[ref-14] Jurjus A, Eid A, Kattar SAl, Zeenny MN, Gerges-Geagea A, Haydar H, Hilal A, Oueidat D, Matar M, Tawilah J, Hussein IH, Schembri-Wismayer P, Cappello F, Tomasello G, Leone A, Jurjus RA (2015). Inflammatory bowel disease, colorectal cancer and type 2 diabetes mellitus: The links. BBA Clinical.

[ref-15] Kelsen JR, Russo P, Sullivan KE (2019). Early-onset inflammatory bowel disease. early-onset inflammatory bowel disease. Immunology and Allergy Clinics of North America.

[ref-16] Lau C, Thibodeaux JR, Hanson RG, Rogers JM, Grey BE, Stanton ME, Butenhoff JL, Stevenson LA (2003). Exposure to perfluorooctane sulfonate during pregnancy in rat and mouse. II: postnatal evaluation. Toxicological Sciences.

[ref-17] Liang X, Xie G, Wu X, Su M, Yang B (2019). Effect of prenatal PFOS exposure on liver cell function in neonatal mice. Environmental Science and Pollution Research International.

[ref-18] Liu X, Zhu Y, Liu T, Xue Q, Tian F, Yuan Y, Zhao C (2020). Exploring toxicity of perfluorinated compounds through complex network and pathway modeling. Journal of Biomolecular Structure and Dynamics.

[ref-19] Lucioli J, Pinton P, Callu P, Laffitte J, Grosjean F, Kolf-Clauw M, Oswald IP, Bracarense AP (2013). The food contaminant deoxynivalenol activates the mitogen activated protein kinases in the intestine: interest of ex vivo models as an alternative to in vivo experiments. Toxicon.

[ref-20] Mahid SS, Minor KS, Soto RE, Hornung CA, Galandiuk S (2006). Smoking and inflammatory bowel disease: a meta-analysis. Mayo Clinic Proceedings.

[ref-21] Miller FW, Alfredsson L, Costenbader KH, Kamen DL, Nelson LM, Norris JM, Roos AJD (2012). Epidemiology of environmental exposures and human autoimmune diseases: findings from a National Institute of Environmental Health Sciences Expert Panel Workshop. Journal of Autoimmunity.

[ref-22] Ng SC, Shi HY, Hamidi N, Underwood FE, Tang W, Benchimol EI, Panaccione R, Ghosh S, Wu JCY, Chan FKL, Sung JJY, Kaplan GG (2018). Worldwide incidence and prevalence of inflammatory bowel disease in the 21st century: a systematic review of population-based studies. Lancet.

[ref-23] Olsen GW, Burris JM, Ehresman DJ, Froehlich JW, Seacat AM, Butenhoff JL, Zobel LR (2007). Half-life of serum elimination of perfluorooctanesulfonate, perfluorohexanesulfonate, and perfluorooctanoate in retired fluorochemical production workers. Environmental Health Perspectives.

[ref-24] Ordas I, Eckmann L, Talamini M, Baumgart DC, Sandborn WJ (2012). Ulcerative colitis. Lancet.

[ref-25] Qazi MR, Xia ZL, Bogdanska J, Chang SC, Ehresman DJ, Butenhoff JL, Nelson BD, DePierre JW, Abedi-Valugerdi M (2009). The atrophy and changes in the cellular compositions of the thymus and spleen observed in mice subjected to short-term exposure to perfluorooctanesulfonate are high-dose phenomena mediated in part by peroxisome proliferator-activated receptor-alpha (PPARalpha). Toxicology.

[ref-26] Steenland K, Zhao L, Winquist A (2015). A cohort incidence study of workers exposed to perfluorooctanoic acid (pfoa). Occupational and Environmental Medicine.

[ref-27] Steenland K, Zhao L, Winquist A, Parks C (2013). Ulcerative colitis and perfluorooctanoic acid (pfoa) in a highly exposed population of community residents and workers in the mid-Ohio valley. Environmental Health Perspectives.

[ref-28] Suo CX, Fan ZQ, Zhou L, Qiu J (2017). Perfluorooctane sulfonate affects intestinal immunity against bacterial infection. Scientific Reports.

[ref-29] UNEP (2016). The Stockholm Convention-National Implementation Plans- Addressing COP 4 amendments.

[ref-30] Wang G, Lu J, Li S, Liu Z, Chang H, Xie C (2018). Pollution levels and risk assessment of perfluoroalkyl acids (PFAAs) in beef muscle and liver from southern Xinjiang. Environmental Science and Pollution Research International.

[ref-31] Wang G, Sun SS, Wu XB, Yang SR, Wu YM, Zhao JX, Zhang H, Chen W (2020). Intestinal environmental disorders associate with the tissue damages induced by perfluorooctane sulfonate exposure. Ecotoxicology and Environmental Safety.

[ref-32] Wang R, Wang R, Niu X, Cheng Y, Shang X, Li Y, Li S, Liu X, Shao J (2019). Role of astrocytes-derived d-serine in PFOS-induced neurotoxicity through NMDARs in the rat primary hippocampal neurons. Toxicology.

[ref-33] Wen LL, Lin CY, Chou HC, Chang CC, Lo HY, Juan SH (2016). Perfluorooctanesulfonate mediates renal tubular cell apoptosis through ppargamma inactivation. PLOS ONE.

[ref-34] Xie SW, Wang TY, Liu SJ, Jones KC, Sweetman AJ, Lu YL (2013). Industrial source identification and emission estimation of perfluorooctane sulfonate in China. Environment International.

[ref-35] Xu YY, Li Y, Scott K, Lindh CH, Jakobsson K, Fletcher T, Ohlsson B, Andersson EM (2020). Inflammatory bowel disease and biomarkers of gut inflammation and permeability in a community with high exposure to perfluoroalkyl substances through drinking water. Environmental Research.

[ref-36] Zhang L, Niu J, Wang Y, Shi J, Huang Q (2014). Chronic effects of PFOA and PFOS on sexual reproduction of freshwater rotifer Brachionus calyciflorus. Chemosphere.

[ref-37] Zhang LM, Rimal BP, Nichols RG, Tian Y, Smith PB, Hatzakis E, Chang SC, Butenhoff JL, Peters JM, Patterso AD (2009). Perfluorooctane sulfonate alters gut microbiota-host metabolic homeostasis in mice. Toxicology.

[ref-38] Zheng L, Dong GH, Jin YH, He QC (2009). Immunotoxic changes associated with a 7-day oral exposure to perfluorooctanesulfonate (PFOS) in adult male C57BL/6 mice. Archives of Toxicology.

