# Supplementary material for: Perfluorooctane sulfonate exerts inflammatory bowel disease-like intestinal injury in rats
Source: PeerJ. 2021 Jan 8;9:e10644. doi: 10.7717/peerj.10644 (PMC7798615; doi:10.7717/peerj.10644)
Supplement: Supplemental Information 3 [file peerj-09-10644-s003.dox]

**Supplementary material**

**Perfluorooctane sulfonate exerts inflammatory bowel disease-like intestinal injury in rats**

Hai Liang^1^, Miao Yang^2^, Cheng Zeng^1^, Wei Wu^1^, Liying Zhao^3^, Yu Wang^4^

1. Department of Pharmacy, The People’s Hospital of Bozhou, Bozhou, Anhui Province, China

2. Department of Neurology, The People’s Hospital of Bozhou, Bozhou, Anhui Province, China

3. Department of Pharmacy, The People’s Hospital of Deqing County, Huzhou, Zhejiang Province, China

4. Department of pharmacy, Zhejiang Integrated Traditional and Western Medicine Hospital, Hangzhou, Zhejiang Province, China

Corresponding Author:

Yu wang^4^

208 Huancheng East Road, Hangzhou, Zhejiang Province, 310003, China.

Email address: wangyuyxb@163.com

Co-corresponding author:

Liying Zhao^3^

120 Yingxi South Road, Wukang Town, Deqing County, Huzhou, Zhejiang Province, 313299, China.

Email address: 302838619@qq.com

**Supplementary Materials & Methods**

*Animals.* Male Sprague-Dawley rats (220 ± 5 g) were purchased from the Laboratory Animal Center of Zhejiang Province (Hangzhou, China). All rats were housed in an environmentally-controlled room (22- 26 ℃, 12 h light-dark cycle, relative humidity 60%), and had free access to standard chow and water. Animals received human care in accordance with the National Institutes of Health Guide for the Care and Use of Laboratory Animals. The animal experiments were accredited by the Ethics Committee of Laboratory Animal Care and Welfare, Zhejiang Academy Medical Sciences, with the proved number 2018-142.

*Experimental protocols.* PFOS was purchased from Sigma Aldrich company (MO, USA), and dissolved in dimethylsulfoxide (DMSO) to the concentration of 100 mg/ml as stock solution. After a one-week adjustable feeding period, the rats were assigned to three groups of 10 rats: two experimental groups and one control group. Rats in the experimental groups were administrated with PFOS by gavage every other day. The concentrations of PFOS were 1 mg/kg and 10 mg/kg. PFOS was diluted by normal saline (NS). Rats in the control group received an equal volume of NS. On the 15th day after PFOS exposure, half rats of three groups were anesthetized (S-ketamine 100 mg/kg, and diazepam 1.5 mg/kg) and sacrificed. Blood, the duodenum, and the jejunum were harvested for biochemical determination or histology. On the 28th day after PFOS exposure, the remaining rats were sacrificed and tissues were collected.

*Blood biochemical.* Blood samples were collected from aorta abdominalis in anesthetized rats. Samples were standing at room temperature (RT) for 1 h, at 4 ℃ for 2 h, which was subsequently centrifuged at 3,000 g for 10 min. The supernatant was harvested and stored at -80 ℃ for blood biochemical index determination. The serum amyloid A (SAA) protein and high sensitivity C reactive protein (hsCRP) were quantified by commercial enzyme-linked immunosorbent assay (ELISA) kits (Uscn Life Science, Wuhan, China) according to the manufacturer’s instructions.

*Histopathological analysis.* The proximal duodenum and proximal jejunum were obtained from three groups for histopathological analysis. Samples were fixed in 4% formalin with phosphate buffer solution (PBS), and were embedded in paraffin. Two 5 μm slides of each rats were cut and stained by hematoxylin and eosin (H&E) for analysis. Photos were picked up at 100 magnifications by a light microscope (Leica Microsystems, Wetzlar, Germany). Intestinal villus was captured at 100 magnifications, and the height of villus was assessed.

Histopathological score of intestinal tissues were performed based on the appearance and severity of lesions according to previously described (Lucioli et al., 2013). The tissue score used a scale of 0-12 points, where numbers of crypts and villus, the height of villus, the morphological change of enterocytes, the extent of villus coalescence, and pathological injury of tissues including autolysis, edema, apoptosis and necrotic debris were referred. The score in regard to morphological and injury data was implemented by two blinded observers.

**References**

**Lucioli J, Pinton P, Callu P, Laffitte J, Grosjean F, Kolf-Clauw M, Oswald IP, Bracarense AP. 2013.** The food contaminant deoxynivalenol activates the mitogen activated protein kinases in the intestine: interest of ex vivo models as an alternative to in vivo experiments. *Toxicon*, **66**:31-6 DOI 10.1016/j.toxicon.2013.01.024.

SFigure1 Effect of PFOS exposure on inflammatory markers and histological lesions in the proximal duodenum and proximal jejunum of rats. Rats were administrated with PFOS by gavage once two days for 15 days or 28 days. The doses of PFOS were 0 mg/kg (normal saline), 1 mg/kg and 10 mg/kg. After PFOS treatment, blood and intestinal tissues of rats were collected for biochemical determination and H&E staining respectively. The high sensitivity C reactive protein (hsCRP; A) and serum amyloid A (SAA) protein (B) were measured by ELISA assay. (C-H) Representative images of the villi from the duodenum and jejunum were shown. Bar 100 μm, 100×. (I) Villi height were analyzed. (J) Lesion scores of the duodenum and the jejunum were assessed. Values are mean ± SD, N = 5/group. **P* < 0.05.
